# Immaturity of the neuromuscular junction in spinal muscular atrophy mouse models

**DOI:** 10.3389/fncel.2026.1795130

**Published:** 2026-03-27

**Authors:** Lucía Tabares, Andrea Fuentes-Moliz, Raquel Cano, Rocío Ruiz, Saravanan Arumugam

**Affiliations:** 1 Department of Physiology and Biophysics, School of Medicine, University of Seville, Seville, Spain; 2 San Isidore University Centre, University Pablo de Olavide, Seville, Spain; 3 Institute of Biomedicine of Seville (IBiS), Virgen del Rocío University Hospital/CSIC/University of Seville, Seville, Spain; 4 Department of Biochemistry and Molecular Biology, School of Pharmacy, University of Seville, Seville, Spain

**Keywords:** active zones, calcium channels, motor neurondevelopment, neuromuscular junction, spinal muscular atrophy, synaptic maturation, synaptic vesicles

## Abstract

Spinal muscular atrophy (SMA) is caused by deficiency of the survival motor neuron (SMN) protein and is classically defined by degeneration of lower motor neurons. Extensive evidence from mouse models and human tissue demonstrates that dysfunction at the neuromuscular junction (NMJ) emerges early and precedes overt denervation. Here, we review structural, molecular, and functional studies showing that SMA NMJs fail to complete key postnatal maturation programmes that normally scale presynaptic release capacity to muscle growth and increasing functional demand. SMA motor terminals retain multiple features of developmental immaturity, including reduced active zone number, limited synaptic vesicle pool extension, altered cytoskeletal organisation, incomplete molecular specialization, and impaired recruitment of functional release sites, resulting in constrained neurotransmitter release and reduced presynaptic reserve. These defects are highly muscle- and region-specific and preferentially affect vulnerable motor units. We propose a conceptual framework in which delayed and incomplete NMJ maturation increases susceptibility to superimposed degenerative processes, ultimately leading to synaptic destabilisation and denervation. This integrated view reconciles early synaptic dysfunction with later neurodegeneration and has important implications for understanding SMA pathogenesis, identifying sensitive biomarkers, and optimizing the timing and targets of therapeutic intervention.

## 1 Introduction

Spinal muscular atrophy (SMA) is an autosomal recessive neuromuscular disorder due to mutations in the survival motor neuron 1 (*SMN1*) gene and characterised by muscle weakness, progressive atrophy and, in severe forms, respiratory failure and early death (Lefebvre et al., 1995; Wirth et al., 2020). The disease is caused by reduced levels of the SMN protein, a ubiquitously expressed protein with essential roles in RNA metabolism, including transcription, splicing, and mRNA transport and translation (Lefebvre et al., 1995; Pellizzoni et al., 1998; Burghes and Beattie, 2009; Lauria et al., 2020). Although SMA has traditionally been classified as a lower motor neuron disease, it is now well recognised that SMN deficiency affects multiple components of the sensorimotor system as well as several peripheral tissues (Kariya et al., 2008; Hamilton and Gillingwater, 2013).

Within the sensorimotor circuit, SMN deficiency disrupts two major synaptic nodes: proprioceptive excitatory inputs onto motor neurons and motor neuron synapses onto skeletal muscle fibres at the NMJ (Mentis et al., 2011; Ling et al., 2010). Alterations at both sites emerge early in disease models and contribute to circuit dysfunction (Mentis et al., 2011; Fletcher et al., 2017; Torres-Benito et al., 2011). Among these, the NMJ represents a particularly sensitive synapse, as it must undergo rapid structural growth and functional refinement during early postnatal life, a developmental window in which SMN levels are especially critical (Kariya et al., 2008; Murray et al., 2008; Gogliotti et al., 2012). Consistent with this vulnerability, studies in mouse models and human tissue have shown that NMJ abnormalities appear early, often before overt denervation or motor neuron loss, and display pronounced muscle- and region-specific patterns (Kariya et al., 2008; Kong et al., 2009; Murray et al., 2015; Torres-Benito et al., 2012; Ling et al., 2012; Woschitz et al., 2022).

At the same time, independent works have demonstrated that SMA motor neurons exhibit impaired motor axon development and maturation (Kong et al., 2021; Kong et al., 2023), early axonal stress and transport defects, leading to abnormal distribution of cytoskeletal elements and presynaptic components in distal axons and nerve terminals (Rossoll et al., 2003; Murray et al., 2008; Franco-Espín et al., 2022). These findings indicate that NMJ pathology in SMA emerges in a cellular context marked by both disrupted axonal and synaptic development and dysfunction.

In this review, we focus on the NMJ and synthesise evidence that SMA motor terminals do not complete key postnatal maturation programmes required to scale synaptic structure and function to muscle growth and increasing physiological demand. We place particular emphasis on presynaptic organisation and neurotransmitter release, drawing extensively on work from our laboratory and others. By organising the literature around postnatal presynaptic scaling, this review aims to clarify how early developmental defects shape synaptic vulnerability and set the stage for later destabilisation and denervation. We propose that delayed and incomplete postnatal maturation of the NMJ in SMA creates a state of functional fragility that precedes and predisposes synapses to later destabilisation and degeneration.

## 2 Postnatal maturation of the neuromuscular junction: scaling synaptic release to growth and demand

**Figure 1:**
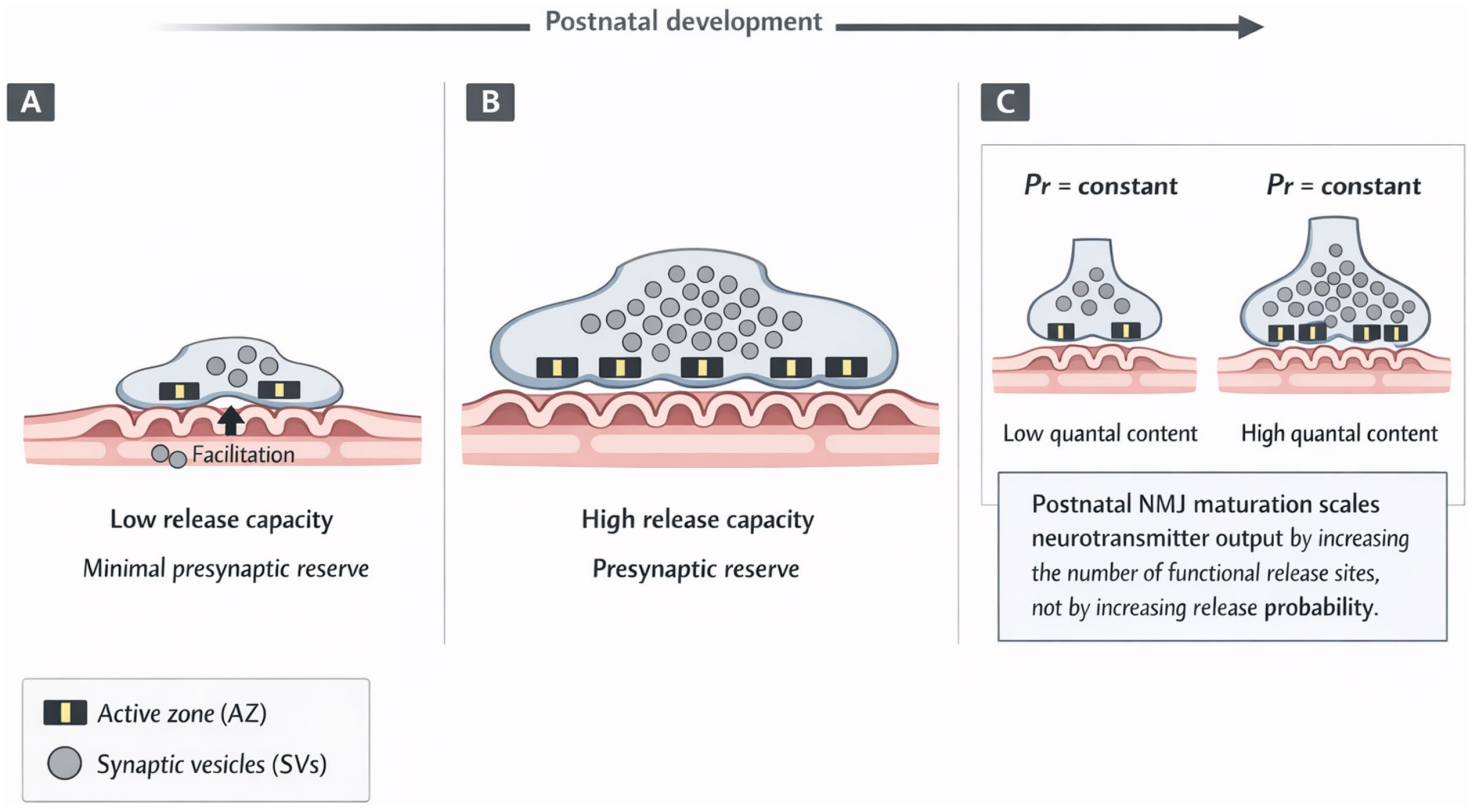
Failure of postnatal pre- and post-synaptic scaling at neuromuscular junctions. Schematic representation of how normal neuromuscular junction (NMJ) maturation scales presynaptic and postsynaptic specializations to accommodate increasing muscle size and functional demand during postnatal development, and how this process is impaired in SMA. Schematics are based on quantitative structural and functional analyses described in Sections 2–6. **(A)** Early postnatal NMJ. Motor nerve terminals are small and contain few active zones (AZs) and limited synaptic vesicle (SV) pools. Postsynaptic specializations are shallow and sparsely folded, resulting in low overall response despite preserved basal neurotransmission. **(B)** Mature NMJ. During postnatal development, motor terminals expand in proportion to muscle growth, increasing AZ number and SV pool size while maintaining relatively stable release probability at individual release sites. In parallel, postsynaptic folds deepen and become more complex, supporting reliable neuromuscular transmission and the establishment of presynaptic functional reserve. **(C)** Core principle. Postnatal NMJ maturation enhances quantal output primarily through the addition of functional release sites and coordinated postsynaptic expansion, rather than by increasing release probability at pre-existing sites.

The NMJ is among the largest and most powerful chemical synapses in the nervous system, yet much of its functional maturation occurs after birth. During early postnatal life, muscle fibres undergo rapid growth, motor behavior becomes increasingly demanding, and patterns of activity diversify. Successful NMJ maturation, therefore, requires coordinated increase of presynaptic release (Figure 1) in proportion to muscle growth and increasing physiological demand (Sanes and Lichtman, 1999; Slater, 2017). Throughout this review, “presynaptic immaturity” refers to delayed and incomplete postnatal maturation, in which developmental programmes are initiated but fail to scale synaptic structure and function to adult levels.

From this perspective, postnatal NMJ maturation can be described as a scaling process. Motor terminals must increase the number of functional release sites and available synaptic vesicles while maintaining appropriate control of release probability to preserve reliability and prevent depletion. This coordinated presynaptic expansion establishes synaptic reserve, defined as the ability to sustain transmission during prolonged or high-demand activity.

### 2.1 Developmental constraints on presynaptic growth

During embryonic stages, nascent NMJs are relatively small and functionally simple (Figure 1A). Motor terminals contain few active zones, limited synaptic vesicle pools, and exhibit low reliability and strong facilitation. Following birth, NMJs enter a period of rapid presynaptic growth, in which active zone number and synaptic vesicle pool size increase in parallel with terminal growth (Figure 1B).

Crucially, this process is achieved primarily through the addition of functional release sites rather than by increasing release probability at individual sites (Tejero et al., 2016). This strategy allows quantal output to increase by an order of magnitude while preserving a high safety factor. Achieving this balance requires coordinated regulation of cytoskeletal remodelling, vesicle trafficking, active zone biogenesis, and calcium channel organisation (Urbano et al., 2002; Cano et al., 2012; Cano et al., 2013; Cano and Tabares, 2016).

Importantly, these maturation programmes are not uniform across all motor units. Muscles differ in fibre-type composition, activation patterns, and biomechanical roles, and consequently display distinct developmental timelines. The postnatal period therefore represents a window during which presynaptic growth must be precisely matched to local functional requirements.

### 2.2 Synapse elimination and terminal growth as functional refinement mechanisms

One of the most prominent events of postnatal NMJ development is the elimination of polyinnervation. Although often viewed as a wiring refinement process, synapse elimination also acts as a refinement-selection mechanism. As supernumerary inputs are removed, the remaining terminal undergoes extensive growth to occupy the full postsynaptic territory.

This expansion is accompanied by the addition of new active zones and growth of synaptic vesicle pools, effectively concentrating release into a single highly specialised terminal. Synapse elimination and terminal growth are therefore integral components of presynaptic scaling, ensuring that function is matched to muscle size and demand (Sanes and Lichtman, 1999).

### 2.3 Functional maturation and the emergence of synaptic reserve

As presynaptic structure expands, NMJ function becomes increasingly reliable and precisely regulated. Quantal content rises steeply during postnatal development, largely through recruitment of additional functional release sites, as schematically illustrated in Figure 1C. In parallel, short-term plasticity profiles evolve, reflecting growth of the readily releasable pool and tighter presynaptic organisation (Urbano et al., 2002; Tejero et al., 2016).

Together, these changes establish synaptic reserve, enabling mature NMJs to sustain reliable transmission during prolonged or high-frequency activity.

### 2.4 Implications for vulnerability in SMA

The requirement for rapid, precisely regulated presynaptic growth places exceptional demands on motor terminals during early postnatal life. Disruption of any component of this maturation programme would be expected to limit synaptic reserve and constrain adaptability. These developmental requirements provide a framework for interpreting the structural abnormalities observed at SMA motor nerve terminals.

## 3 Structural evidence for impaired presynaptic maturation at SMA neuromuscular junctions

During postnatal development, motor nerve terminals enlarge in parallel with muscle growth through coordinated increases in synaptic vesicle pools, active zone number, and cytoskeletal organisation (Figure 1). In SMA, multiple lines of evidence indicate selectively impaired postnatal maturation in vulnerable motor units (Kariya et al., 2008; Kong et al., 2009; Murray et al., 2008; Ruiz et al., 2010; Woschitz et al., 2022). As a result, NMJs operate closer to maximal capacity, with limited ability to accommodate increased demand. Importantly, the spatial organisation and regular spacing of remaining active zones, together with preserved release probability and calcium sensitivity, argue against predominant primary degenerative loss and instead are more consistent with incomplete postnatal addition of presynaptic specialisations. This framework underlies the detailed structural, molecular, and functional abnormalities described in the following sections.

### 3.1 Synaptic vesicle pools and distribution

**Figure 2:**
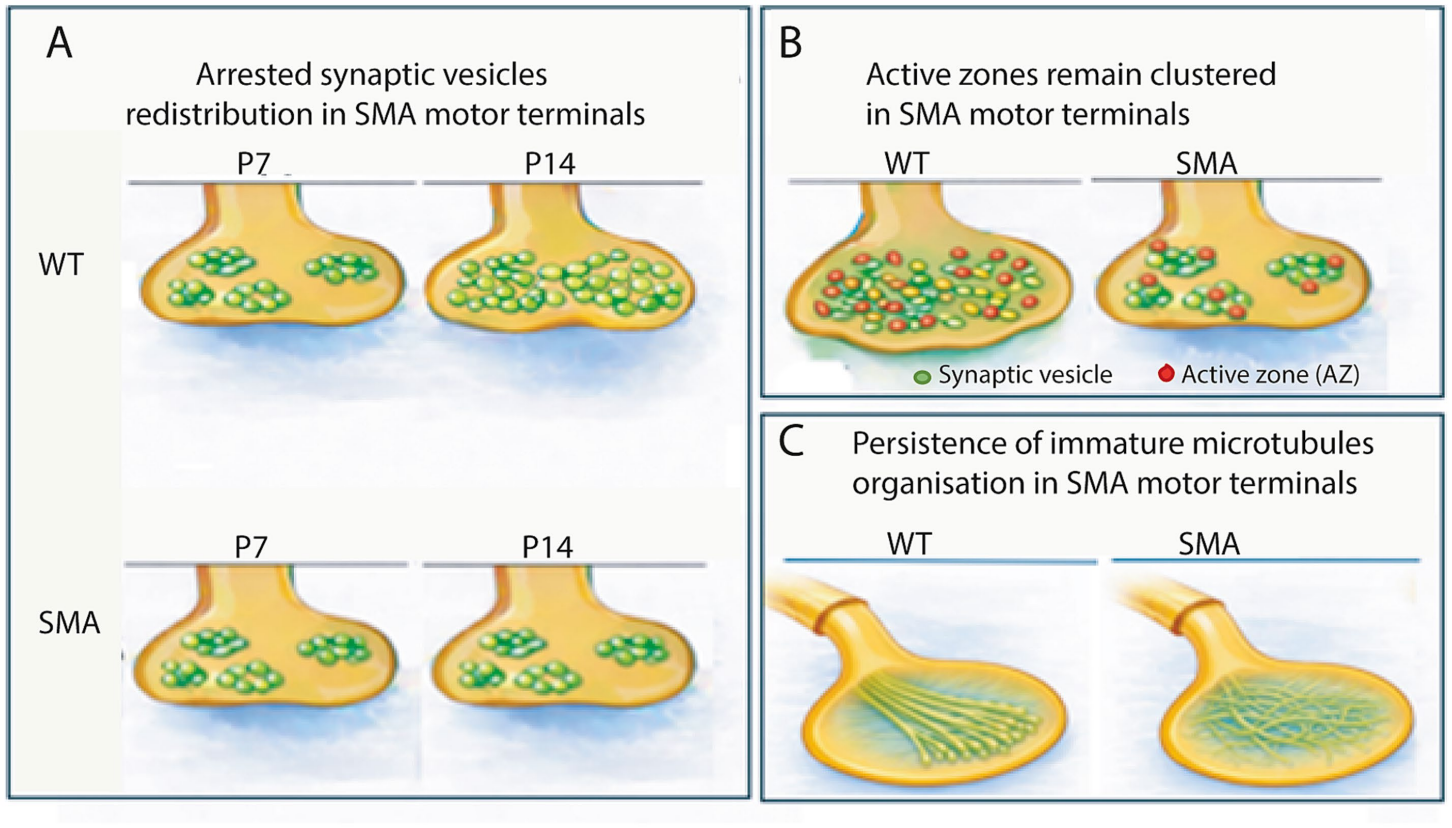
Schematic representation of impaired presynaptic maturation at the NMJ in SMA summarizing the requirement of SMN for proper organisation of synaptic vesicles, active zones, and the microtubule cytoskeleton at motor nerve terminals. The model is inspired by experimental observations reported in Torres-Benito et al. (2011; *PLoS One*), Figures 1, 4, and 7, and is presented here as an original, redrawn conceptual illustration. **(A)** Synaptic vesicle organisation. In control NMJs (upper), synaptic vesicle (SV) pools expand and consolidate during postnatal maturation. In SMA motor terminals (lower), SV pools remain smaller, fragmented, and spatially restricted relative to postsynaptic growth. **(B)** Active zone scaling. In control terminals (left), the number of active zones (AZs, red spheres) scales with terminal size and distributed across the presynaptic membrane. In SMA (right), AZ number fails to scale as in controls, resulting in reduced AZ density and uneven distribution. **(C)** Cytoskeletal organisation. During normal maturation, presynaptic cytoskeletal remodelling produces organised microtubule architecture that supports terminal growth (left). In SMA (right), motor terminals retain immature cytoskeletal features, including disorganised microtubules and persistence of neurofilament accumulations.

In control NMJs, synaptic vesicle clusters progressively enlarge and merge during postnatal maturation, forming dense vesicle-rich regions that sustain neurotransmitter release (Figure 2A, upper panels). In contrast, SMA motor terminals retain smaller, fragmented synaptic vesicle clusters (Figure 2A, lower panels) that remain spatially restricted despite ongoing muscle growth (Kong et al., 2009; Torres-Benito et al., 2011; Dachs et al., 2011).

Deficits in synaptic vesicle pool enlargement are highly muscle- and region-specific. Motor terminals innervating selectively vulnerable muscles, such as the transversus abdominis, exhibit pronounced reductions in vesicle cluster size and density, whereas relatively resistant muscles, including rostral regions of the levator auris longus, show milder or delayed alterations. Within individual muscles, regional gradients in vesicle organisation correlate with patterns of structural vulnerability, reinforcing the link between impaired presynaptic maturation and selective susceptibility (Torres-Benito et al., 2011).

### 3.2 Active zone number and organisation

Active zones are the discrete presynaptic specialisations where voltage-gated calcium channels, vesicle docking, and fusion are tightly coupled. Immunolabelling and electron microscopy studies demonstrate a significant reduction in active zone number relative to postsynaptic area at SMA NMJs during the period of postnatal growth (Torres-Benito et al., 2011; Dachs et al., 2011). At developmental stages when control NMJs display a homogeneous distribution of active zones across the presynaptic terminal, SMA motor endings exhibit clustered and uneven active zone organisation. This defect closely parallels the failure to add synaptic vesicles, indicating a coordinated impairment of presynaptic scaling mechanisms. Quantitative nearest-neighbour analyses of active zone interdistances reveal that the remaining active zones are regularly spaced, whereas large regions of the terminal lack active zones altogether (Figure 2B). This spatial pattern is inconsistent with purely random loss of pre-existing active zones and is more consistent with incomplete addition of new active zones.

Active zone deficits in SMA are not uniform across all muscles. NMJs innervating selectively vulnerable muscles, such as the transversus abdominis or intercostals, display the most pronounced reductions in active zone number. In contrast, relatively resistant muscles show milder or delayed alterations (Torres-Benito et al., 2011). Even within individual muscles, regional differences in active zone density correlate with gradients of structural impairment, reinforcing the concept of muscle- and region-specific disruption of presynaptic maturation.

### 3.3 Cytoskeletal organisation in SMA motor nerve terminals

Proper maturation of presynaptic terminals requires extensive cytoskeletal remodelling to support axonal growth, vesicle trafficking, active zone assembly and stabilisation of synaptic structure. In SMA, motor nerve terminals retain multiple cytoskeletal features characteristic of immature developmental states.

One of the most consistent hallmarks of SMA NMJs is the persistence of neurofilament loops within the motor nerve terminal. Neurofilament loops are commonly observed at early postnatal stages in control terminals but are normally eliminated as terminals mature. In SMA mouse models, these loops persist beyond the normal developmental window and are particularly prominent in vulnerable muscles (Torres-Benito et al., 2011). Their presence reflects impaired cytoskeletal remodelling and limited radial growth of the motor axon.

Microtubule organisation is also altered in SMA motor terminals. During normal maturation, microtubules transition from a dispersed arrangement to more organised, bundled structures that support axonal transport and terminal growth. In SMA NMJs, microtubules retain a web-like appearance (Figure 2C), characteristic of immature terminals (Torres-Benito et al., 2011). Such an organisation is consistent with delayed cytoskeletal maturation during a critical postnatal window.

In addition to abnormalities in neurofilaments and microtubules, accumulating evidence indicates that regulation of the actin cytoskeleton is disrupted in SMA. Actin dynamics are essential for presynaptic terminal growth, synaptic vesicle mobilisation, and the stabilisation of active zones. SMN interacts, directly and indirectly, with multiple actin-regulatory proteins, and reduced SMN levels result in altered actin polymerisation and impaired growth cone and axonal dynamics in motor neurons (Bowerman et al., 2009; Rossoll et al., 2003). In SMA models, disrupted actin organisation is associated with impaired axon outgrowth, reduced terminal complexity, and abnormal synaptic development, supporting delayed presynaptic maturation. Moreover, dysregulation of actin-binding proteins such as profilin IIa and plastin 3 (PLS3) further supports a role for defective actin remodelling in constraining terminal development and contributing to presynaptic vulnerability during postnatal development (Ackermann et al., 2013; Bowerman et al., 2009; Oprea et al., 2008; Jablonka and Schäfer, 2024; Hennlein et al., 2023).

Taken together, the persistence of neurofilament loops, immature microtubule organisation, and impaired actin remodelling define a characteristic cytoskeletal phenotype of SMA motor nerve terminals during early postnatal ages.

## 4 Postsynaptic and muscle contributions to NMJ vulnerability in SMA

While presynaptic defects are a primary focus of this review, abnormalities in postsynaptic and muscle compartments modulate the efficacy and stability of neuromuscular transmission and shape how presynaptic immaturity is functionally expressed.

### 4.1 Delayed postsynaptic endplate maturation

Normal postnatal NMJ maturation (Figure 2) involves extensive remodelling of the postsynaptic membrane, including expansion of endplate area, increased structural complexity, and formation of deep junctional folds aligned with presynaptic active zones (Sanes and Lichtman, 1999; Slater, 2017). These specialisations increase the safety factor of neuromuscular transmission.

In SMA mouse models and human tissue, postsynaptic maturation is delayed, particularly in vulnerable muscles. Endplates are smaller and less complex (Figure 2), and the developmental switch from the embryonic *γ*-subunit to the adult *ε*-subunit of the acetylcholine receptor (AChR) is delayed at early postnatal stages (Kariya et al., 2008; Murray et al., 2008; Martínez-Hernández et al., 2009; Lee et al., 2011). These features reduce postsynaptic efficiency and decrease the safety margin for transmission.

Beyond delayed maturation, recent work indicates that SMN is required to preserve endplate integrity. Disruption of the SMN–U7 snRNP pathway reduces agrin expression in muscle, leading to fragmentation and destabilisation of AChR clusters (Tisdale et al., 2022). Thus, in SMA, postsynaptic defects reflect not only developmental delay but also impaired molecular mechanisms that maintain synaptic architecture.

### 4.2 Skeletal muscle fibre maturation and retrograde signalling

Muscle fibre growth and differentiation are also perturbed in SMA (Berciano et al., 2024; Ottoboni et al., 2026). Reduced fibre size, delay in the transition from neonatal to adult isoforms of proteins such as ryanodine receptors, sodium channels (Na_v_1.4), and myosin heavy chain have been documented in SMA mouse models and human tissue (Kong et al., 2009; Martínez-Hernández et al., 2009; Lee et al., 2011; Boyer et al., 2013; Lee et al., 2025). These alterations are muscle-specific and parallel patterns of NMJ vulnerability.

Because muscle-derived signals regulate presynaptic growth, active zone stabilisation and synaptic vesicle organisation, delayed muscle maturation is expected to alter retrograde signalling during postnatal development. Such changes modify the synaptic environment in which presynaptic maturation occurs and can influence NMJ stability.

## 5 SMN-dependent RNA regulation links SMN deficiency to presynaptic development

Beyond its canonical role in small nuclear ribonucleoprotein assembly, SMN directly participates in RNA metabolism within motor axons, providing a mechanistic link between SMN deficiency and altered synaptic development at the NMJ. SMN localises to neurites, axons, and motor nerve terminals as a core component of axonal ribonucleoprotein complexes, where it supports neuronal differentiation and synaptic maturation by enabling the transport and local availability of specific mRNAs at developing motor nerve terminals (Fan and Simard, 2002; Rossoll et al., 2003; Fallini et al., 2011; Dombert et al., 2014; Kye et al., 2014; Franco-Espín et al., 2022). In line with this framework, reduced SMN levels in SMA models disrupt axonal mRNA localisation and translation early, before overt denervation or motor neuron loss, indicating a primary defect in synaptic development rather than a secondary consequence of degeneration (Rossoll et al., 2003; Murray et al., 2008; Gabanella et al., 2016).

A key element of the synaptic maturation pathway is the interaction between SMN and neuronal RNA-binding proteins (RBPs), including hnRNP R, HuD, and IMP1 (Rossoll et al., 2002; Dombert et al., 2014; Fallini et al., 2014; Donlin-Asp et al., 2017). These RBPs bind and stabilise transcripts upregulated during late stages of NMJ maturation and promote their axonal localisation (Akten et al., 2011; Dombert et al., 2014). In SMA, reduced SMN levels impair RBP-dependent mRNA stabilisation and localisation, leading to selective depletion of maturation-associated transcripts at motor nerve terminals (Fallini et al., 2016).

Notably, many RBP-regulated transcripts encode proteins involved in active zone assembly, synaptic vesicle release, cytoskeletal regulation, and calcium-dependent signalling. Disruption of this RNA regulatory pathway likely lies upstream of the molecular and structural abnormalities observed at SMA NMJs.

## 6 Functional consequences of impaired presynaptic scaling in SMA

The structural and molecular abnormalities described above are accompanied by pronounced deficits in neuromuscular transmission. Electrophysiological analyses across multiple muscles and developmental stages demonstrate that SMA NMJs are unable to generate or sustain the levels of neurotransmitter release required during postnatal growth and increasing motor demand.

### 6.1 Reduced quantal content and muscle-specific vulnerability

In selectively vulnerable muscles, such as the transversus abdominis (TVA), tibialis anterior (TA), extensor digitorum longus (EDL), and the caudal region of the levator auris longus (LALc) quantal content is significantly reduced at early postnatal stages. In contrast, release is preserved in less vulnerable muscles or muscular regions such as in the rostral part of the LAL (LALr) muscle (Table 1).

**Table 1:** Quantal content at the neuromuscular junction in spinal muscular atrophy models during postnatal maturation.

SMA mouse model	Postnatal age	Muscle	Quantal content decrease	References
SMNΔ7	P9-10	TA	43%	Kong et al. (2009)
	P7-8	TVA	55%	Ruiz et al. (2010)
	P7-8	LAL rostral	0%	Ruiz et al. (2010)
	P7-8	LAL caudal (C2)	51%	Ruiz et al. (2010)
	P12-14	EDL	25%	Ling et al. (2010)
Taiwanese	P6-8	TVA	29%	López-Manzaneda et al. (2021)
Smn2B/−	P15	TVA	52%	Her et al. (2026)

**Figure 3:**
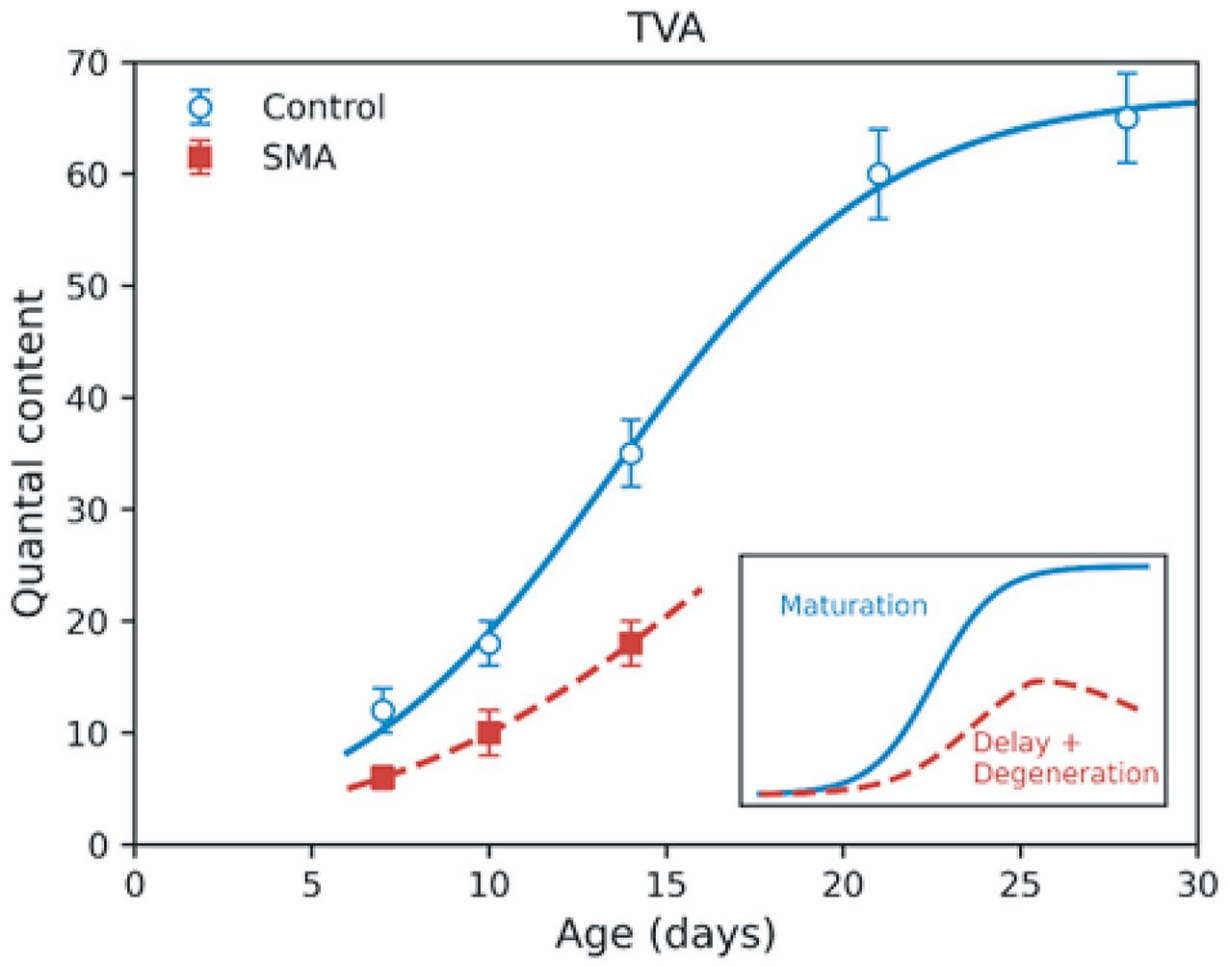
Delayed and incomplete maturation of presynaptic neurotransmitter release at vulnerable NMJs in SMA. This figure is an illustrative synthesis based on published electrophysiological datasets. In control mice, quantal content increases during postnatal development and reaches a stable plateau, consistent with normal presynaptic scaling and establishment of synaptic reserve. In SMA mice, the developmental increase in quantal content is delayed and truncated, and mature release levels are not attained before disease progression limits further synaptic growth. Data points shown are derived from experimental measurements in the transversus abdominis (TVA) muscle of the SMAΔ7 mouse model, as reported in Ruiz et al. (2010), Tejero et al. (2016), and Fuentes-Moliz et al. (2023). These studies examined postnatal stages spanning early symptomatic to pre-symptomatic periods and collectively support the altered developmental trajectory illustrated. Inset: Schematic comparison of normal presynaptic maturation (blue) versus delayed and incomplete maturation in SMA (red), illustrating constrained functional scaling of neurotransmitter release.

Although release capability increases during the second postnatal week, mature levels are not attained before disease progression constrains synaptic growth. Figure 3 provides a synthesis of datasets to illustrate the altered developmental trajectory of quantal content in SMA (Ruiz et al., 2010; Tejero et al., 2016; Fuentes-Moliz et al., 2023). Consistent with this framework, the postnatal trajectory of quantal content at vulnerable NMJs is delayed and truncated in SMA (Figure 3, inset).

### 6.2 Calcium dependence of neurotransmitter release

Evoked neurotransmitter release at SMA NMJs remains strongly dependent on extracellular calcium across a broad concentration range. Neither the apparent calcium cooperativity of release nor the extracellular calcium concentration required for half-maximal release differs significantly between control and SMA NMJs (Tejero et al., 2016).

However, maximal neurotransmitter output is reduced, and saturation is reached at lower absolute levels of quantal content. These findings indicate that release probability at individual functional release sites and the core calcium-sensing machinery are largely preserved, while total release is constrained.

### 6.3 Reduced recruitment of functional release sites and limited readily releasable pool

In SMA motor terminals, increases in extracellular calcium fail to recruit additional functional release sites beyond those already active at lower concentrations. As a result, maximal quantal content remains constrained despite preserved calcium sensitivity. Direct estimates of the readily releasable pool obtained using high-frequency stimulation trains reveal a marked reduction in pool size at SMA NMJs (Torres-Benito et al., 2011; Tejero et al., 2016).

### 6.4 Short-term synaptic plasticity reflects limited presynaptic reserve

Short-term synaptic plasticity profiles at SMA NMJs closely resemble those of immature control synapses. Facilitation is preserved or exaggerated, while depression develops rapidly during sustained stimulation, indicating limited presynaptic reserve (Kong et al., 2009; Ruiz et al., 2010; Tejero et al., 2016).

### 6.5 Integrative functional readouts across SMA models and patients

Across different SMA mouse models, synaptic recordings consistently show a similar phenotype: reduced quantal content, rapid synaptic depression during repetitive stimulation, and preserved mEPP amplitude, indicating a primary limitation in presynaptic release. These deficits are most evident in vulnerable muscles and early symptomatic stages, identifying reduced recruitment of functional release sites and limited synaptic reserve as core functional features.

At the motor unit level, *in vivo* electrophysiological recordings in severe SMNΔ7 mice demonstrate early and progressive reductions in compound muscle action potential (CMAP) amplitudes and motor unit number estimates (MUNE) during the first two postnatal weeks, consistent with declining motor unit output prior to extensive denervation (Arnold et al., 2014). Needle electromyography reveals spontaneous fibrillation potentials at symptomatic stages, indicating active denervation. Restoration of SMN expression via antisense oligonucleotide preserves MUNE and partially or fully restores CMAP amplitudes (M-wave and H-wave), validating these measures as translational biomarkers of motor unit dysfunction (Arnold et al., 2016; Bogdanik et al., 2015; Simon et al., 2025).

In patients with SMA, reduced CMAP amplitudes, decreased motor unit numbers, and spontaneous EMG abnormalities correlate with clinical severity and disease progression. Importantly, SMN-restoring therapies increase CMAP amplitudes, particularly when administered early, indicating that impaired neuromuscular transmission contributes substantially to functional decline and remains partially reversible (Finkel et al., 2017; Simon et al., 2025).

These findings indicate that the limitation in neurotransmitter output observed at individual SMA NMJs scales to impaired motor unit performance *in vivo*. The next question, therefore, is which molecular mechanisms constrain this presynaptic growth during postnatal development.

## 7 Molecular determinants of impaired presynaptic maturation and reserve in SMA

The structural and functional limitations of SMA NMJs are accompanied by altered regulation of key presynaptic proteins. Several molecular programmes that normally mature during the early postnatal period either do not complete or proceed aberrantly in SMA, thereby altering active zone composition, synaptic vesicle organisation, and calcium–release coupling.

### 7.1 Developmental regulation of presynaptic calcium channel usage

At mature mammalian NMJs, evoked neurotransmitter release is mediated predominantly by P/Q-type (CaV2.1) voltage-gated calcium channels. During normal postnatal development, an early contribution of N-type (CaV2.2) channels is progressively replaced by P/Q-type channels, stabilising calcium–release coupling (Urbano et al., 2002).

In SMA mouse models, this developmental transition is largely achieved without alteration of release cooperativity. Pharmacological analyses indicate that P/Q-type channels remain the dominant calcium source for release, yet their relative contribution is modestly reduced compared with controls, with a residual N-type component persisting beyond the normal developmental window (Tejero et al., 2016). Despite this alteration, calcium cooperativity and apparent calcium sensitivity remain unchanged.

### 7.2 Synaptotagmin isoform switching and calcium sensing

Postnatal maturation of the NMJ also involves a switch in the expression of synaptotagmin isoforms. Synaptotagmin-1 (Syt1) and synaptotagmin-2 (Syt2) both serve as fast calcium sensors for synchronous vesicle fusion, but apparently Syt2 supports faster kinetics and improved performance during high-frequency activity (Geppert et al., 1994; Pang et al., 2006).

At mammalian NMJs, postnatal functional maturation is accompanied by a developmental shift toward Syt2 predominance (Tejero et al., 2016), similar to that observed at another giant synapse, the calyx of Held, in the central nervous system (Kochubey et al., 2016). In SMA mouse models, this synaptotagmin switch is delayed or incomplete in a muscle-specific manner. Functionally vulnerable muscles (abdominal wall muscles) display reduced Syt2 expression together with persistence of Syt1, whereas more resistant muscles (LALr, diaphragm) maintain comparatively higher levels of both calcium sensors (Tejero et al., 2016).

### 7.3 Molecular alterations of functional release sites in motor nerve terminals

Accumulating evidence indicates that motor nerve terminals in SMA exhibit specific molecular alterations and that SMN contributes to the regulation of synaptic function.

Synaptotagmins interact closely with synaptic vesicle protein 2 (SV2), a family of vesicle membrane proteins essential for normal calcium-triggered exocytosis (Chang and Südhof, 2009; Stout et al., 2019). At the NMJ, the SV2B isoform is selectively enriched and associated with fast synchronous release.

In SMA motor terminals, SV2B expression is selectively reduced in vulnerable muscles, closely paralleling the decrease in Syt2 levels, whereas other presynaptic proteins such as syntaxin-1B or synaptotagmin-7 are relatively preserved (Tejero et al., 2016). This selective molecular signature distinguishes vulnerable from resistant NMJs.

Recent work has also identified alterations in the presynaptic levels of the priming factor Munc13-1 at NMJs in SMA mice (Moradi et al., 2015). Replacement of the endogenous Munc13-1 3′UTR with a heterologous 3′UTR restored axonal mRNA localisation and increased Munc13-1 protein levels in cultured SMA motoneurons. *In vivo*, conditional knock-in mice expressing the modified Munc13-1 allele showed increased NMJ Munc13-1 levels, a higher proportion of fully innervated endplates, increased spinal motoneuron counts, improved motor performance, and extended survival compared with SMA controls. These findings demonstrate that altered Munc13-1 mRNA localization and reduced presynaptic Munc13-1 characterize SMA motor terminals, and that restoring Munc13-1 levels ameliorates structural and functional disease phenotypes *in vivo*.

Consistent with a broader alteration of the vesicle fusion machinery, SMN deficiency has also been reported to impair SNARE complex assembly in motor nerve terminals (Kim et al., 2023). In that study, a modifier variant in the constitutive chaperone HSPA8 (Hspa8^G470R^) restored SNARE assembly to levels comparable to controls.

Together, these findings delineate a set of presynaptic molecular alterations affecting vesicle priming, fusion, and release-site organisation in SMA motor terminals.

## 8 Cellular mechanisms driving motor nerve terminal degeneration in SMA

**Figure 4:**
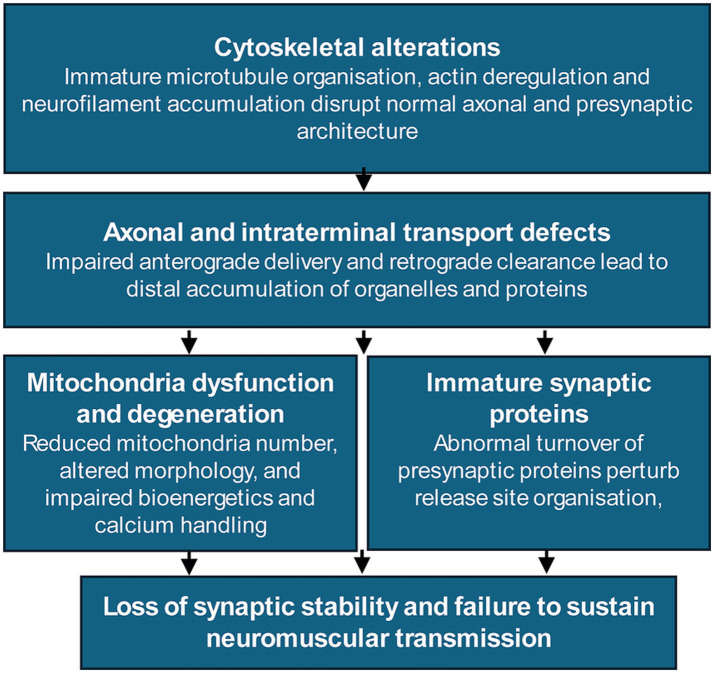
Cellular mechanisms contributing to motor nerve terminal destabilisation in SMA. Multiple, interacting cellular defects converge to compromise the structural and functional stability of motor nerve terminals. Cytoskeletal alterations, including neurofilament accumulation, microtubule disorganisation, and actin dysregulation, disrupt axonal architecture and presynaptic integrity. These changes impair axonal transport, leading to defective anterograde delivery and retrograde clearance with distal accumulation of organelles and presynaptic cargo. Mitochondrial dysfunction at nerve terminals further limits bioenergetic support and calcium handling, while mislocalisation and abnormal turnover of synaptic proteins, including SMN, perturb release site organisation and maintenance. Together, these interconnected processes result in loss of synaptic stability and failure to sustain neuromuscular transmission.

While delayed and incomplete postnatal maturation imposes early functional constraints on SMA NMJs, multiple studies demonstrate that motor nerve terminals also engage active degenerative processes that progressively destabilise synaptic structure, as shown in Figure 4. These abnormalities are detectable early and preferentially affect distal axons and nerve terminals.

SMA motor neurons display reduced axonal calibre and axonal transport dysfunctions (Figure 4). Reduced SMN levels impair the assembly and trafficking of ribonucleoprotein complexes and motor protein-associated cargoes, leading to defective delivery of presynaptic components at distal axons and nerve terminals (Rossoll et al., 2003; Murray et al., 2008). In addition, defective clearance by retrograde transport produces protein accumulation in the terminal, mainly neurofilaments and SMN, which become progressively more pronounced as the disease advances (Kariya et al., 2008; Murray et al., 2008; Lee et al., 2011; Franco-Espín et al., 2022).

Mitochondrial abnormalities represent an additional contributor to motor nerve terminal degeneration and muscle dysfunction in SMA (James et al., 2021). Altered mitochondrial distribution, reduced mitochondrial content, impaired mitochondrial bioenergetics, and reduced intramitochondrial calcium (Figure 4) have been described in SMA models (Kariya et al., 2008; Kong et al., 2009; Torres-Benito et al., 2011; Murray et al., 2015; López-Manzaneda et al., 2021; Franco-Espín et al., 2022). Given the high energetic demands of synaptic vesicle recycling and neurotransmitter release, mitochondrial dysfunction is expected to exacerbate synaptic stress.

Together, axonal transport failure, cytoskeletal disorganisation, abnormal protein accumulation, and mitochondrial dysfunction define a constellation of degenerative cellular processes that compromise the stability of SMA motor nerve terminals. These interacting cellular alterations at SMA motor nerve terminals are summarised schematically in Figure 4.

## 9 Developmental immaturity and degeneration as interacting determinants of NMJ vulnerability in SMA

NMJ pathology in SMA emerges from the interaction between incomplete postnatal synaptic maturation and superimposed degenerative stress, whose relative contributions evolve over time. Importantly, these processes are not independent; limited presynaptic growth may increase structural and metabolic stress at motor nerve terminals, thereby sensitising them to degenerative cascades.

As development proceeds, cellular stressors associated with reduced SMN levels, including impaired axonal transport, abnormal protein accumulation, cytoskeletal destabilisation and mitochondrial dysfunction, progressively challenge synaptic stability (Figure 4). In NMJs already operating with limited reserve, these stressors are more likely to destabilise presynaptic architecture, disrupt vesicle availability and impair release site integrity.

Differences in developmental timing, activity patterns, and presynaptic growth requirements across motor units are likely to interact with SMN deficiency to shape selective vulnerability, although the relative contribution of each factor remains to be fully defined. Systematic comparative analyses across multiple SMA mouse models demonstrate that neuronal vulnerability follows divergent patterns depending on motor neuron pool identity and disease severity (Woschitz et al., 2022). Some motor units undergo early and pronounced degeneration, whereas others display relative structural and functional resilience despite comparable SMN deficiency. These findings reinforce the view that NMJ immaturity and synaptic destabilisation do not reflect a uniform motor neuron phenotype but instead emerge within a landscape of pool-specific susceptibility shaped by intrinsic factors. This integrated framework reconciles early synaptic impairment with subsequent motor neuron loss within a unified pathogenic model.

## 10 SMA disease modifiers and synaptic resilience

Although SMN deficiency is the primary cause of SMA, disease severity is not determined exclusively by SMN2 copy number. Several genetic modifiers influence phenotypic variability, indicating that synaptic vulnerability can be buffered by endogenous mechanisms. Among these, PLS3 was the first characterised modifier with direct relevance to NMJ function.

PLS3 is an actin-bundling protein initially identified as protective in asymptomatic SMA carriers (Oprea et al., 2008). In SMA models, increased PLS3 expression improves axonal growth and ameliorates NMJ structural and functional defects (Ackermann et al., 2013). Because actin remodelling is essential for presynaptic terminal growth, vesicle mobilisation and active zone stabilisation, PLS3 is thought to counteract cytoskeletal dysregulation associated with SMN deficiency (Bowerman et al., 2009).

Within the framework proposed in this review, PLS3 may enhance presynaptic scaling by reinforcing actin-dependent structural support during postnatal NMJ growth. Actin stabilisation through PLS3 could facilitate vesicle trafficking and endocytic recycling, processes required to sustain transmitter release under functional demand (Wu and Chan, 2022).

Also recently, a potent genetic modifier of SMA has been identified: a variant of the heat shock protein Hspa8 (Hspa8^G470R^). Hspa8 is constitutively expressed and enriched at synapses. In SMAΔ7 mice, expression of Hspa8^G470R^ markedly ameliorated disease severity, including restoration of neurotransmitter release at the NMJ and a substantial extension of survival (Kim et al., 2023). Mechanistically, this modifier enhanced correct SMN2 splicing, resulting in modest increases in full-length SMN protein, and restored synaptic SNARE complex assembly, which is disrupted under conditions of low SMN (Kim et al., 2023).

Together, these findings support a model in which disease modifiers such as PLS3 and Hspa8^G470R^ buffer the failure of presynaptic scaling in SMA through distinct but convergent mechanisms: stabilisation of actin-dependent synaptic architecture and vesicle cycling in the case of PLS3, and improved SMN-dependent RNA processing and SNARE complex restoration in the case of Hspa8^G470R^. Targeting these complementary pathways may augment the success of SMN-restoring therapies by enhancing NMJ resilience during critical windows of postnatal maturation.

## 11 Conclusions and perspectives

Accumulating evidence from SMA mouse models and human studies indicates that structural and molecular abnormalities at the NMJ constrain function in vulnerable motor units. Multiple presynaptic maturation processes, including active zone growth, synaptic vesicle pool growth, cytoskeletal remodelling and molecular specialisation of the release machinery, remain incomplete in vulnerable motor units, constraining neurotransmitter release and limiting synaptic adaptability. Although much of the mechanistic evidence derives from mouse models, available analyses of human SMA tissue reveal parallel delays in NMJ and muscle maturation, supporting the relevance of these developmental mechanisms to human disease.

These findings have important implications for understanding disease progression and therapeutic intervention. Parameters that reflect NMJ maturation, such as active zone density, synaptic vesicle organisation, calcium–release coupling and short-term synaptic plasticity, may serve as sensitive biomarkers of disease state and treatment efficacy, complementing traditional measures of motor neuron survival or muscle strength.

From a translational perspective, the developmental timing of intervention emerges as a critical determinant of outcome. Therapies that restore SMN levels or directly support presynaptic maturation and scaling mechanisms are likely to be most effective when applied early, during periods of active synaptic growth and refinement. Later interventions may stabilise existing synapses or slow degenerative progression but may be less capable of fully restoring synaptic capacity once critical developmental windows have passed.

Clinical experience with SMN-restoring therapies demonstrates that NMJ pathology is at least partially reversible when treatment is initiated early. In patients, increases in compound muscle action potential (CMAP) amplitudes following nusinersen or risdiplam indicate improved motor unit output, particularly with presymptomatic intervention (Finkel et al., 2017; Darras et al., 2021). Similarly, systemic AAV9-mediated SMN restoration in SMA mouse models enhances NMJ innervation and improves neuromuscular transmission (Dominguez et al., 2011). These findings support the view that impaired motor unit performance reflects, to a significant extent, dysfunctional neuromuscular transmission rather than irreversible motor neuron loss. However, while global electrophysiological measures demonstrate functional recovery, detailed analyses at the single-synapse level remain limited. Whether presynaptic release capacity, active zone number, and synaptic reserve are fully normalised after SMN restoration have not yet been comprehensively defined in animal models. Incorporating NMJ-specific functional parameters into therapeutic evaluation will therefore be essential to determine the extent of true synaptic rescue.

Finally, the concept that disrupted synaptic maturation predisposes NMJs to later instability may extend beyond SMA to other neuromuscular and neurodevelopmental disorders in which synaptic growth, scaling, or refinement are compromised. Further investigation into the mechanisms governing postnatal synaptic maturation will not only advance understanding of SMA pathogenesis but may also reveal broader principles of synaptic vulnerability and resilience.
